# Intelligent Reflecting Surface–Assisted Wireless Secret Key Generation against Multiple Eavesdroppers

**DOI:** 10.3390/e24040446

**Published:** 2022-03-23

**Authors:** Ya Liu, Kaizhi Huang, Xiaoli Sun, Shaochuan Yang, Liang Wang

**Affiliations:** 1 Wireless Communication Technology Office, Information Engineering University, Zhengzhou 450002, China; liuya2014@zua.edu.cn (Y.L.); lgdxsunxiaoli@sina.com (X.S.); yangsc@zua.edu.cn (S.Y.); jym@ndsc.com.cn (L.W.); 2 School of Intelligent Engineering, Zhengzhou University of Aeronautics, Zhengzhou 450015, China

**Keywords:** Intelligent reflecting surface, physical layer secret key generation, semi-definite relaxation, convex–concave procedure

## Abstract

In this paper, we propose an improved physical layer key generation scheme that can maximize the secret key capacity by deploying intelligent reflecting surface (IRS) near the legitimate user aiming at improving its signal-to-noise ratio (SNR). We consider the scenario of multiple input single output (MISO) against multiple relevant eavesdroppers. We elaborately design and optimize the reflection coefficient matrix of IRS elements that can improve the legitimate user’s SNR through IRS passive beamforming and deteriorate the channel quality of eavesdroppers at the same time. We first derive the lower bound expression of the achievable key capacity, then solve the optimization problem based on semi-definite relaxation (SDR) and the convex–concave procedure (CCP) to maximize the secret key capacity. Simulation results show that our proposed scheme can significantly improve the secret key capacity and reduce hardware costs compared with other benchmark schemes.

## 1. Introduction

The tremendous growth in extensive connectivity, ubiquity, and diversity of the sixth generation (6G) wireless communication networks will result in unpredictable security threats [1]. Traditionally, data confidentiality is guaranteed by the high-layer encryption mechanism based on computational complexity. However, the key distribution for massive devices is complicated. Furthermore, the public key cryptography may be completely cracked by the emerging quantum computers in the future [2]. As a powerful supplement to the existing high-layer encryption, the physical layer key generation (PKG) technology [3] makes use of the inherent characteristics of wireless channels (i.e., temporal variation, uniqueness, and reciprocity). The PKG extracts the key from the channel characteristics, which are considered as the natural shared random source [4,5,6]. It is information-theoretically secure and lightweight and is considered to be one of the enhanced technologies of 6G secure communication [7].

Key generation usually consists of four main steps: acquisition of shared random source, quantization, information reconciliation, and privacy amplification. First, two legitimate users, namely Alice and Bob, transmit pilots to each other in turn, and they estimate the channels between them to obtain correlated channel measurements. Then, these measurements are converted into binary bits by using quantization algorithms [8]. Next, information reconciliation is used to correct inconsistent bits between Alice and Bob [9]. Finally, the privacy amplification phase is employed to eliminate possible information leakage in the previous phases [10]. However, the channel estimation can have low accuracy due to the poor quality of the received signal in the acquisition of shared random source, introducing a high key inconsistency rate and low key entropy after quantization. In [11], the authors consider the scenario that the base station uses the maximum ratio transmission (MRT) to maximize the SNR of the received signal, but this scheme results in limited improvement of the SNR. We also use preprocessing methods to remove the non-reciprocity caused by noise. However, part of the channel characteristic information will be abandoned, which reduces the key generation rate (KGR), increases the complexity of baseband algorithm processing, and introduces poor real-time performance.

Recently, intelligent reflecting surfaces (IRSs) that can flexibly control the electromagnetic characteristics of wireless channels have received much attention and are considered as a significant technology for 6G [12,13]. An IRS is typically constructed by using a printed circuit board (PCB), which consists of a reflective unit layer, a copper plate, and a control circuit board. The reflecting elements are equally spaced on a two-dimensional plane and are composed of full metal sheets on the bottom layers and metal patches on the top layers of the PCB dielectric substrates [14]. Moreover, a semiconductor device can vary the impedance of the reflecting element by controlling its biasing voltage so that the electromagnetic response can be dynamically tuned in real time without changing the geometrical parameters [15]. IRSs can achieve real-time configuration of various parameters of the reflected electromagnetic wave, including phase, amplitude, and polarization direction, and can artificially control the radio wave transmission. In addition, IRSs can smartly program and reconstruct electromagnetic waves in real time to customize the wireless environment with passive characteristics. IRSs are used to preprocess the wavefront of reflected electromagnetic waves; specifically, the polarization direction of the received signal is adjusted to match the polarization of the antenna of the legitimate receiver [16], which enhances the received signal strength.

Complex algorithm design is not required in our IRS-assisted PKG, which effectively reduces the key inconsistency and improves the SNR of the legitimate receiver and the key entropy. Research on IRS-assisted PKG has emerged recently. In Ref. [17], the authors design an IRS reflecting coefficient matrix to maximize the SNR received by the legitimate user in the single input single output (SISO) network. An IRS is utilized to introduce artificial randomness for boosting the secret key rate by adjusting the IRS phase switching to modify the propagation environment in [18,19,20]. In Ref. [19], the authors optimized the switching states of the IRS elements to maximize the secret key rate. A limited number of IRS elements were turned on, which corresponded to the largest variances of the IRS channels. However, the analysis of the secret key rate was greatly simplified, because the eavesdroppers’ channels were assumed to be independent of the legitimate users’. In Ref. [20], the authors further use IRSs and Wi-Fi equipment to build a prototype system to achieve any adjustable key rate. To sum up, IRSs can be utilized to improve the SNR or enhance the randomness as an artificial random source in the research of PKG. An IRS is used to improve the SNR in this paper, and the relationship between the number of IRS elements and transmitting antennas is not further explored in the current research. IRS-assisted PKG in multi-antenna networks remains unexplored; however, this is more relevant for the actual communication scenarios.

In this paper, we mainly consider an IRS-assisted MISO system model against multiple non-colluding eavesdroppers (non-colluding eavesdropping is practical in the IoT scenario, where nodes exist independently and do not cooperate. Partially colluding and fully colluding eavesdroppers will be discussed in Section 4), who gather around the legitimate user, targeting to obtain private information. We deploy an IRS near the legitimate user and elaborately design an IRS reflection coefficient matrix that can improve the legitimate user’s SNR through IRS passive beamforming, and thereby the legitimate user’s channel estimation accuracy is improved. At the same time, multiple eavesdroppers around the legitimate user, after IRS tuning, offset the reflected signals with the direct link signals from the base station, therefore deteriorating the channel quality and effectively reducing information leakage. In the presence of multiple passive eavesdroppers, we formulate the secret key capacity of the system and optimize the IRS reflection coefficient to maximize the secret key capacity under the constraint that the modulus square of the IRS reflection coefficient is less than one. Due to the non-convexity of this optimization problem, we employ SDR and CCP to solve it. Simulation results show that our scheme significantly improves the secret key capacity compared with MRT and IRS with random phase shifts.

The rest of the paper is organized as follows. Section 2 describes the system model discussed in this paper and signal representation. In Section 3, we discuss the IRS-assisted secret key rate and derive the expression of key generation capacity. In Section 4, the proposed optimization scheme and solution are presented. Section 5 presents the simulation results. Section 6 draws the conclusions.

## 2. System Model and Signal Representation

**Figure 1:**
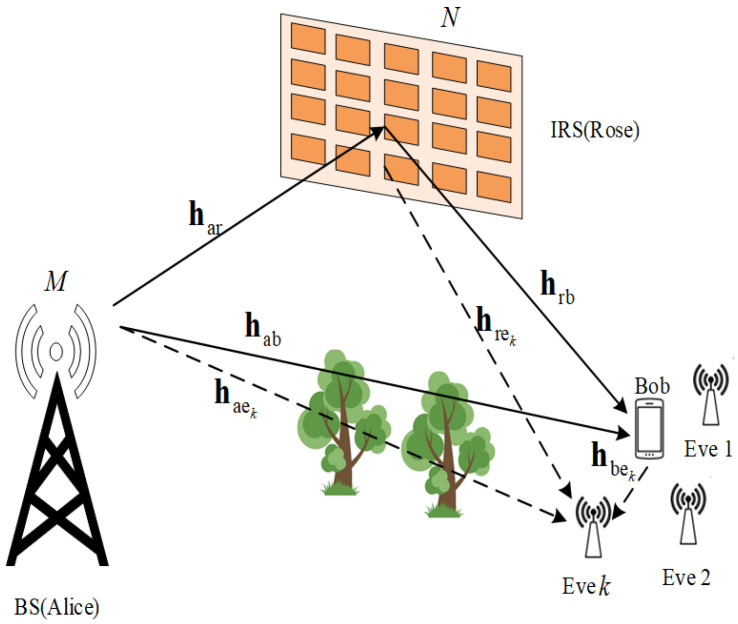
System model for IRS-assisted network against multiple Eves.

As shown in Figure 1, a base station Alice and a single antenna user Bob are legitimate communication nodes aiming to extract the shared secret keys from their reciprocal wireless channel with the assistance of an IRS (Rose). K non-colluding eavesdroppers (Eves) attempt to acquire the transmitted information generated by Alice and Bob based on their own channel observations. All the eavesdroppers are non-colluding, meaning that each eavesdropper performs independent decoding to retrieve signals without relying on other eavesdroppers [21]. Suppose that all Eves are equipped with a single antenna, while the number of antennas at Alice are denoted by M. Rose is composed of N reflecting elements deployed in a uniform planar array (UPA), with X vertical elements and Y horizontal elements, and can change its reflection coefficients to minimize the information leaked to Eve.

### 2.1. Channel Model

The equivalent channels from Alice to Rose, Bob, and Eve k (the k-th eavesdropper) are denoted by $h_{\mathrm{ar}}\in {\mathbb{C}}^{N\times M}$, $h_{\mathrm{ab}}\in {\mathbb{C}}^{1\times M}$, and $h_{{\mathrm{ae}}_{k}}\in {\mathbb{C}}^{1\times M}$, respectively, while the equivalent channels from Rose to Bob and Eve k are denoted by $h_{\mathrm{rb}}\in {\mathbb{C}}^{1\times N}$ and $h_{{\mathrm{re}}_{k}}\in {\mathbb{C}}^{1\times N}$, respectively.

Let $\Phi =\mathrm{diag}(\beta_{1}e^{j\theta_{1}},\beta_{2}e^{j\theta_{2}},\cdots ,\beta_{N}e^{j\theta_{N}})$ represent the diagonal phase-shifting matrix of Rose, where in its main diagonal, $\beta_{n}\in [0,1]$ and $\theta_{n}\in [0,2\pi )$ denote the amplitude and phase shift on the incident signal by Rose’s n-th element, for n=1,…,N. The reflecting coefficient vector of Rose $v={\left[v_{1},v_{2},\cdots ,v_{N}\right]}^{T}$ is defined, where the n-th element of v is $v_{n}=\beta_{n}e^{j\theta_{n}}$, which is the n- th diagonal element of Φ. 

We consider the case where the light of sight (LOS) path between the base station and Bob/Eve is blocked, and the Rayleigh fading model is considered due to plenty of scatters in this wireless channel, i.e., each element of $h_{\mathrm{ab}}$ and $h_{{\mathrm{ae}}_{k}}$ is independent and identically distributed in the complex Gaussian distribution of zero mean and variance, $\sigma_{h_{\mathrm{ab}}}{}^{2}$ and $\sigma_{h_{{\mathrm{ae}}_{k}}}{}^{2}$, respectively. Considering the base station and IRS are usually set up at a higher altitude, we assume that only the LOS path exists between Alice and Rose. The BS-to-IRS channel can be modeled as in [22]:

(1) $$h_{\mathrm{ar}}=\eta_{1}\alpha_{\mathrm{IRS}}\left(\theta_{\mathrm{IRS}},\gamma_{\mathrm{IRS}}\right)\alpha_{\mathrm{BS}}{}^{H}(\varphi_{\mathrm{BS}}),\;$$

where $\eta_{1}\sim \mathrm{CN}\left(0,10^{-0.1\mathrm{PL}(d_{\mathrm{ar}})}\right)$ denotes the complex gain of the BS-to-IRS channel, and $\alpha_{\mathrm{IRS}}\left(\theta_{\mathrm{IRS}},\gamma_{\mathrm{IRS}}\right)$ and $\alpha_{\mathrm{BS}}\left(\varphi_{\mathrm{BS}}\right)$ are the normalized array steering vectors of IRS and BS. They are denoted by

(2) $$\alpha_{\mathrm{IRS}}\left(\theta_{\mathrm{IRS}},\gamma_{\mathrm{IRS}}\right)={\left[1,\cdots ,e^{j2\pi \frac{d_{R}}{\lambda }\left[\left(x-1\right)\sin\theta_{\mathrm{IRS}}\sin\gamma_{\mathrm{IRS}}+\left(y-1\right)\cos\gamma_{\mathrm{IRS}}\right]},\cdots ,e^{j2\pi \frac{d_{R}}{\lambda }\left[\left(X-1\right)\sin\theta_{\mathrm{IRS}}\sin\gamma_{\mathrm{IRS}}+\left(Y-1\right)\cos\gamma_{\mathrm{IRS}}\right]}\right]}^{T}\;$$

(3) $$\alpha_{\mathrm{BS}}\left(\varphi_{\mathrm{BS}}\right)={\left[1,\cdots ,e^{j2\pi \frac{d_{B}}{\lambda }\left(M-1\right)\sin\varphi_{\mathrm{BS}}}\right]}^{T},$$

where $\theta_{\mathrm{IRS}}\in \left[0,\pi \right]$ and $\gamma_{\mathrm{IRS}}\in \left[0,\pi \right]$ represent the elevation and azimuth angles of arrival (AOA) at IRS, respectively, and $\varphi_{\mathrm{BS}}\in \left[0,2\pi \right]$ is the azimuth angle of departure (AOD) from the BS and IRS. λ is the wavelength, and $d_{R}$ and $d_{B}$ denote the element spacing at the IRS and the antenna spacing at the BS, respectively, and in this paper, they are both set as half wavelength, i.e., we have $d_{R}=d_{B}=\frac{\lambda }{2}$. The path loss can be derived as $PL\left(d\right)\left[\mathrm{dB}\right]=PL_{0}+10c\log_{10}\left(d\right)$, where d, $PL_{0}$ and c denote the distance between transmitter and receiver, constant path loss term, and path loss exponent, respectively. For the channels between the IRS and Bob/Eve, LoS components exist in practical implementation. We use Rician fading to model the channel $h_{\mathrm{rb}}$ and $h_{{\mathrm{re}}_{k}}$, which can be denoted by

(4) $$h_{\mathrm{ru}}=\sqrt{\frac{\kappa_{j}}{\kappa_{j}+1}}{\bar{h}}_{\mathrm{ru}}+\sqrt{\frac{1}{\kappa_{j}+1}}{\tilde{h}}_{\mathrm{ru}},u\in \left\{b,e_{k}\right\},j=2,3\;$$

where $\kappa_{2}$ and $\kappa_{3}$ is the Rician κ-factor of $h_{\mathrm{rb}}$ and $h_{{\mathrm{re}}_{k}}$; ${\bar{h}}_{\mathrm{ru}}\in {\mathbb{C}}^{1\times N}$ is the LoS component, which remains unchanged within the channel coherence time; and ${\tilde{h}}_{\mathrm{ru}}\in {\mathbb{C}}^{1\times N}$ is the non-LoS (NLoS) component, whose elements follow the zero-mean complex Gaussian distribution. The LoS component ${\bar{h}}_{\mathrm{ru}}$ is expressed as

(5) $${\bar{h}}_{\mathrm{ru}}=\eta_{j}\alpha^{H}{}_{\mathrm{IRS},u}\left(\varphi_{\mathrm{IRS},u},\omega_{\mathrm{IRS},u}\right),j=2,3$$

where $\alpha_{\mathrm{IRS},u}\left(\varphi_{\mathrm{IRS},u},\omega_{\mathrm{IRS},u}\right)$ is the normalized array response vector of the UPA at IRS, $\varphi_{\mathrm{IRS},u}\in \left[0,\pi \right]$ and $\omega_{\mathrm{IRS},u}\in \left[0,\pi \right]$ denote the elevation and azimuth AOD from IRS to Bob/Eve k, respectively, where $\eta_{2}$ and $\eta_{3}$ are the complex gain of IRS-to-Bob and IRS-to-Eve channels, respectively. To generate the shared secret keys, Alice and Bob will alternatively exchange pilots and perform channel estimations. In the downlink channel probing, the BS broadcasts the orthogonal pilot signal $S_{1}\in {\mathbb{C}}^{M\times T_{1}}$, where $T_{1}$ is the length of pilot, $S_{1}{\left(S_{1}\right)}^{H}=I_{M\times M}$, and I is an M×M identity matrix. Assuming that the transmitting power of the base station (BS) is P, the received signal of Bob or the k-th Eve is given by

(6) $$Y_{u}{}^{DL}=\sqrt{P}\left(h_{\mathrm{ab}}+h_{\mathrm{ru}}\Phi h_{ar}\right)S_{1}+N_{u}{}^{DL}=\sqrt{P}\left(h_{\mathrm{au}}+v^{T}G_{u}\right)S_{1}+N_{u}{}^{DL},u\in \left\{b,e_{k}\right\},$$

where $G_{u}=\mathrm{diag}(h_{\mathrm{ru}})h_{\mathrm{ar}}$ and $N_{b}{}^{DL}$ and $N_{e_{k}}^{DL}$ follow complex additive white Gaussian noise (AWGN) with variance $\sigma_{1}{}^{2}$ and $\sigma_{2,k}{}^{2}$ of each element. Similarly, Bob sends the orthogonal pilot signal $S_{2}\in {\mathbb{C}}^{M\times T_{2}}$ in the uplink channel probing, where $T_{2}$ is the length of pilot and $S_{2}{\left(S_{2}\right)}^{H}=I_{M\times M}$. Bob’s transmit power is also P. Thus Alice and Eve k receive

(7) $$Y_{a}{}^{UL}=\sqrt{P}\left(h_{\mathrm{ba}}+h_{\mathrm{ar}}{}^{T}\Phi h_{\mathrm{rb}}{}^{T}\right)S_{2}+N_{a}{}^{UL}=\sqrt{P}\left(h_{\mathrm{ab}}{}^{T}+G_{b}{}^{T}v\right)S_{2}+N_{a}{}^{UL}$$

(8) $$Y_{e_{k}}{}^{UL}=\sqrt{P}\left(h_{{\mathrm{be}}_{k}}+h_{{\mathrm{re}}_{k}}\Phi h_{\mathrm{rb}}{}^{T}\right)S_{2}+N_{e_{k}}{}^{UL},$$

where $N_{a}{}^{UL}$ is complex AWGN with variance $\sigma_{1}{}^{2}$ of each element due to channel reciprocity, and $N_{e_{k}}{}^{UL}$ follows complex additive white Gaussian noise (AWGN) $\sigma_{3}{}^{2}$ of each element.

Assuming that the sampling interval is adequately small, the channel reciprocity preserves between the bidirectional pilot transmissions within the coherent time. By the least square (LS) estimation, the combined channels at each respective node after normalization can be expressed as

(9) $$\begin{array}{l} Z_{b}{}^{DL}=h_{\mathrm{ab}}+v^{T}G_{b}+N_{b}{}^{{DL}^{\prime }}\\ Z_{a}{}^{UL}=h_{\mathrm{ab}}{}^{T}+G_{b}{}^{T}v+N_{a}{}^{{UL}^{\prime }}\\ Z_{e_{k}}{}^{DL}=h_{{\mathrm{ae}}_{k}}+v^{T}G_{e_{k}}+N_{e_{k}}{}^{{DL}^{\prime }}\\ Z_{e_{k}}{}^{UL}=h_{{\mathrm{be}}_{k}}+h_{{\mathrm{re}}_{k}}\Phi h_{\mathrm{rb}}{}^{T}+N_{e_{k}}{}^{{UL}^{\prime }} \end{array}$$

where the variance of each element in AWGN $N_{b}{}^{{DL}^{\prime }}$ and $N_{a}{}^{{UL}^{\prime }}$ are $\sigma_{1}{}^{2}/P$, and in AWGN $N_{e_{k}}{}^{{DL}^{\prime }}$ and $N_{e_{k}}{}^{{UL}^{\prime }}$ are $\sigma_{2}{}^{2}/P$ and $\sigma_{3}{}^{2}/P$, respectively. After channel probing, BS and UT vectorize the estimated channel matrices $h_{B}=\mathrm{vec}\left(Z_{b}{}^{DL}\right)$ and $h_{A}=\mathrm{vec}\left(Z_{a}{}^{UL}\right)$ to generate the secret key. After employing quantization, information reconciliation and privacy amplification, the BS and UT finally generate the consistent secret key. Similarly, Eves vectorize the estimated channel matrices $h_{E_{k,1}}=\mathrm{vec}\left(Z_{e_{k}}{}^{DL}\right),h_{E_{k,2}}=\mathrm{vec}\left(Z_{e_{k}}{}^{UL}\right)$ to guess the same key.

### 2.2. Spatial Correlation

When the eavesdropper k is located at least several wavelengths away from the legitimate user, the channels from the IRS to them, $h_{\mathrm{rb}}$ and $h_{{\mathrm{re}}_{k}}$ are assumed to be independent with each other [23]. Furthermore, when the number of IRS elements is large, the IRS-induced channels $h_{\mathrm{rb}}\Phi h_{\mathrm{ar}}$ and $h_{{\mathrm{re}}_{k}}\Phi h_{\mathrm{ar}}$ are statistically independent [24]. However, with the rapid development of communication technology, more and more unattended sensor terminals are put into use in the Internet of Things scenarios, and illegal terminals try to get close to legitimate ones, resulting in the difficulty of ensuring the security of legitimate users at all times. When eavesdroppers are located close to the legitimate users, the corresponding channels $h_{\mathrm{rb}}$ and $h_{{\mathrm{re}}_{k}}$ may be correlated. We define the correlation coefficient between them as

(10) $$\rho_{k}=\frac{E\left\{h_{\mathrm{rb}}h_{\mathrm{re}}{{}_{{}_{k}}}^{H}\right\}}{\sqrt{E\left\{h_{\mathrm{rb}}h_{\mathrm{rb}}{}^{H}\right\}E\left\{h_{\mathrm{re}}{}_{{}_{k}}h_{\mathrm{re}}{{}_{{}_{k}}}^{H}\right\}}}$$

where a larger value of $\rho_{k}$ implies a higher correlation between channels of the eavesdropper k and legitimate user. The channel correlation in (10) implies that IRS-induced channels $h_{\mathrm{rb}}\Phi h_{\mathrm{ar}}$ and $h_{{\mathrm{re}}_{k}}\Phi h_{\mathrm{ar}}$ may be correlated. When eavesdroppers have the same ability, the closer Eve k is to Bob, the higher the correlation between them.

The cross-correlation between the channels corresponding to Eve k and Bob can be expressed as

(11) $$\begin{array}{c} R_{k}=E\left\{{\left(h_{\mathrm{ab}}+h_{\mathrm{rb}}\Phi h_{\mathrm{ar}}\right)}^{T}{\left(h_{{\mathrm{ae}}_{k}}+h_{{\mathrm{re}}_{k}}\Phi h_{\mathrm{ar}}\right)}^{*}\right\}\\ \;=E\left\{h_{\mathrm{ar}}{}^{T}\Phi {\bar{h}}_{rb}{}^{T}{\bar{h}}_{{\mathrm{re}}_{k}}{}^{*}\Phi^{H}h_{\mathrm{ar}}{}^{*}\right\}\\ \;=E\left\{h_{\mathrm{ar}}{}^{T}\mathrm{diag}\left({\bar{h}}_{{}_{\mathrm{rb}}}\right)vv^{H}\mathrm{diag}\left({\bar{h}}_{{\mathrm{re}}_{k}}\right)h_{\mathrm{ar}}{}^{*}\right\} \end{array}$$

which will be further analyzed in Section 3 to derive its expression.

## 3. IRS-Assisted Secret Key Rate Analysis

In this section, a closed-form expression of the secret key capacity for a lower bound is derived. We conservatively take the lower bound capacity as the minimum achievable secret key rate. The lower bound of the key capacity [25] can be expressed as

(12) $$C\left(h_{A};h_{B}\|h_{E_{k,1}},h_{E_{k,2}}\right)\geq \max\left\{I\left(h_{A};h_{B}\right)-I\left(h_{A};h_{E_{k,1}},h_{E_{k,2}}\right),I\left(h_{A};h_{B}\right)-I\left(h_{B};h_{E_{k,1}},h_{E_{k,2}}\right)\right\}$$

where I( ; ) denotes the mutual information.

We assume that Alice and Rose know the statistics of the channel state information (CSI) of all channels. This is a recognized assumption in large-scale wireless networks, in which the eavesdropper is an authorized but untrusted user trying to obtain the information of other users [26]. Below, we derive the lower bound of IRS-assisted secret key capacity. Considering the uplink channel estimation at the Eve side is scalar and poses a small threat to information leakage, we only consider the impact of downlink channel estimations, i.e., $h_{E_{k,1}}$ of Eve on key capacity, in this paper. We set $h_{E_{k}}=h_{E_{k,1}}$.The lower bound is expressed as the maximum of $C_{k,1}=I\left(h_{A};h_{B}\right)-I(h_{A};h_{E_{k}})$ and $C_{k,2}=I\left(h_{A};h_{B}\right)-I(h_{B};h_{E_{k}})$, so we derive closed form expressions of $C_{k,1}$ and $C_{k,2}$, then select the maximum value as the final expression. First, we express the first term $C_{k,1}$ as

(13) $$C_{k,1}=I\left(h_{A};h_{B}\right)-I\left(h_{A};h_{E_{k}}\right)=H(h_{A}/h_{E_{k}})-H(h_{A}/h_{B})=\log_{2}\frac{\det(W_{{\mathrm{AE}}_{k}})\det\left(K_{B}{}_{B}\right)}{\det(K_{E_{k}E_{k}})\det(W_{\mathrm{AB}})},$$

where H() is the information entropy, $W_{xy}=E\left\{{\left[h_{x},h_{y}\right]}^{T}[h_{x}{}^{H},h_{y}{}^{H}]\right\}$, $x,y\in \left\{A,B,E_{k}\right\}$, and $K_{xy}=E\left\{h_{x}h_{y}{}^{H}\right\}$ are correlation matrices, and det (.) is the determinant of matrix. Furthermore, we can derive

(14) $$\begin{array}{l} K_{\mathrm{AA}}=E\left\{h_{A}h_{A}{}^{H}\right\}\\ \overset{①}{=}E\left\{h_{\mathrm{ab}}{}^{T}h_{\mathrm{ab}}{}^{*}+h_{\mathrm{ar}}{}^{T}\Phi h_{\mathrm{rb}}{}^{T}h_{\mathrm{rb}}{}^{*}\Phi^{H}h_{\mathrm{ar}}{}^{*}+N_{a}{}^{{UL}^{\prime }}{\left(N_{a}{}^{{UL}^{\prime }}\right)}^{H}\right\}\\ \overset{②}{=}\frac{\kappa_{2}}{\kappa_{2}+1}E\left\{\left(h_{\mathrm{ar}}{}^{T}\Phi {\bar{h}}_{\mathrm{rb}}{}^{T}{\bar{h}}_{\mathrm{rb}}{}^{*}\Phi^{H}h_{\mathrm{ar}}{}^{*}\right)\right\}+\sigma_{A}{}^{2}I\\ \overset{③}{=}\frac{\kappa_{2}}{\kappa_{2}+1}E\left\{\left(h_{\mathrm{ar}}{}^{T}\mathrm{diag}\left({\bar{h}}_{\mathrm{rb}}\right)vv^{H}\mathrm{diag}\left({\bar{h}}_{\mathrm{rb}}{}^{*}\right)h_{\mathrm{ar}}{}^{*}\right)\right\}+\sigma_{A}{}^{2}I\\ \overset{④}{=}Q_{A}\left(v\right)R_{\mathrm{BS}}+\sigma_{A}{}^{2}I \end{array}$$

In Equation (1), we can substitute the expression of $h_{A}$. In Equation (2), we substitute $h_{\mathrm{rb}}$ according to Equation (4), and the mean value of NLOS component ${\tilde{h}}_{\mathrm{rb}}$ is 0. In Equation (4), $h_{\mathrm{ar}}$ and ${\bar{h}}_{\mathrm{rb}}$ according to Equations (1) and (5) are substituted and calculated by matrix multiplication. $\sigma_{A}{}^{2}=\sigma_{h_{\mathrm{ab}}}{}^{2}+\sigma_{1}{}^{2}/P$ and $Q_{A}\left(v\right)$ can be expressed as

(15) $$Q_{A}\left(v\right)=\frac{\kappa_{2}}{\kappa_{2}+1}{\left|\eta_{1}\eta_{2}\right|}^{2}v^{H}\beta^{H}\beta v$$

The vector β is given as

(16) $$\beta =[1,\cdots ,e^{\tau_{x,y}\left(\theta_{\mathrm{IRS}},\gamma_{\mathrm{IRS}}\right)+\tau_{x,y}\left(\varphi_{\mathrm{IRS},B},\omega_{\mathrm{IRS},B}\right)},\cdots ,e^{\tau_{X,Y}\left(\theta_{\mathrm{IRS}},\gamma_{\mathrm{IRS}}\right)+\tau_{X,Y}\left(\varphi_{\mathrm{IRS},B},\omega_{\mathrm{IRS},B}\right)}]$$

where $\tau_{x,y}\left(\theta ,\gamma \right)$ is defined as

(17) $$\tau_{x,y}\left(\theta ,\gamma \right)=e^{j2\pi \frac{d}{\lambda }\left(\left(x-1\right)\sin\theta \sin\gamma +\left(y-1\right)\cos\gamma \right)}.$$

We define matrix $R_{\mathrm{BS}}=E\left\{\left[\alpha_{\mathrm{BS}}{\left(\varphi_{\mathrm{BS}}\right)}^{*}\alpha_{\mathrm{BS}}{\left(\varphi_{\mathrm{BS}}\right)}^{T}\right]\right\}$, the rank of which is equal to 1.

It can also be derived that $K_{\mathrm{BB}}=K_{\mathrm{AA}}=Q_{A}\left(v\right)R_{\mathrm{BS}}+\sigma_{A}{}^{2}I$,$K_{\mathrm{AB}}=K_{\mathrm{BA}}=Q_{A}\left(v\right)R_{\mathrm{BS}}+\sigma_{h_{\mathrm{ab}}}{}^{2}I$.

Similarly, we can get $K_{E_{k}A}=K_{{\mathrm{AE}}_{k}}=E\left\{h_{A}h_{E_{k}}{}^{H}\right\}=R_{k}$, where $R_{k}$ is the cross-correlation between the channels corresponding to Eve k and Bob. We derive $R_{k}$ as

(18) $$K_{E_{k}A}=K_{{\mathrm{AE}}_{k}}=R_{k}=R_{{\mathrm{AE}}_{k}}\left(v\right)R_{\mathrm{BS}}\;$$

where $R_{{\mathrm{AE}}_{k}}\left(v\right)$ and ψ can be denoted as

(19) $$R_{{\mathrm{AE}}_{k}}\left(v\right)=\sqrt{\frac{\kappa_{2}\cdot \kappa_{3}}{\left(\kappa_{2}+1\right)\left(\kappa_{3}+1\right)}}{\left|\eta_{1}\right|}^{2}\eta_{2}\eta_{3}{}^{*}v^{H}\psi \beta v$$

(20) $$\psi ={\left[1,\cdots ,e^{-\tau_{x,y}\left(\theta_{\mathrm{IRS}},\gamma_{\mathrm{IRS}}\right)}\cdot e^{-\tau_{x,y}\left(\varphi_{\mathrm{IRS},E},\omega_{\mathrm{IRS},E}\right)},\cdots \right]}^{T}$$

Similarly, we get $K_{E_{k}E_{k}}=\left\{h_{E_{k}}h_{E_{k}}{}^{H}\right\}=Q_{E_{k}}\left(v\right)R_{\mathrm{BS}}+\sigma_{E_{k}}{}^{2}I$, where $Q_{E_{k}}\left(v\right)=\frac{\kappa_{3}}{\kappa_{3}+1}{\left|\eta_{1}\eta_{3}\right|}^{2}v^{H}\psi^{H}\psi v$, $\sigma_{E_{k}}{}^{2}=\sigma_{h_{{\mathrm{ae}}_{k}}}{}^{2}+\sigma_{2}{}^{2}/P$. Thus, the determinant of matrix terms $W_{{\mathrm{AE}}_{k}}$ and $W_{\mathrm{AB}}$ in (11) can be expressed as

(21) $$\det\left(W_{{\mathrm{AE}}_{k}}\right)=\det\left(\begin{array}{cc} K_{\mathrm{AA}} & K_{{\mathrm{AE}}_{k}}\\ K_{{\mathrm{AE}}_{k}} & K_{E_{k}E_{k}} \end{array}\right)=\det\left(K_{\mathrm{AA}}\right)\cdot \det(K_{E_{k}E_{k}}-K_{{\mathrm{AE}}_{k}}K_{\mathrm{AA}}{}^{-1}K_{{\mathrm{AE}}_{k}})$$

(22) $$\det\left(W_{\mathrm{AB}}\right)=\det\left(K_{\mathrm{AA}}\right)\cdot \det(K_{B}{}_{B}-K_{\mathrm{AB}}K_{\mathrm{AA}}{}^{-1}K_{\mathrm{AB}})$$

We substitute $\det\left(W_{{\mathrm{AE}}_{k}}\right)$, $\det\left(K_{\mathrm{BB}}\right)$, $\det\left(K_{E_{k}E_{k}}\right)$ and $\det\left(W_{\mathrm{AB}}\right)$ into Equation (11) above. Because $Q_{A}\left(v\right),R_{{\mathrm{AE}}_{k}}\left(v\right)$, and $Q_{E_{k}}\left(v\right)$ are all scalars, the ranks of $Q_{A}\left(v\right)R_{BS},R_{{\mathrm{AE}}_{k}}\left(v\right)R_{\mathrm{BS}}$, and $Q_{E_{k}}\left(v\right)R_{\mathrm{BS}}$ are also equal to 1, which means that they all have, at most, one non-zero eigenvalue. Therefore, we can take advantage of this property that the determinant of a determined matrix is equal to the product of all eigenvalues of the matrix.

**Theorem** **1.** *For Eve^k^, the achievable secret key capacity *$C_{k,1}$*in IRS-assisted wireless networks is calculated as*

(23) $$\begin{array}{l} \begin{array}{l} C_{k,1}=\log_{2}\frac{\left(MQ_{E_{k}}\left(v\right)+\sigma_{E_{k}}{}^{2}\right){\left(MQ_{A}\left(v\right)+\sigma_{A}{}^{2}\right)}^{2}-M^{2}R_{{\mathrm{AE}}_{k}}{\left(v\right)}^{2}\left(MQ_{A}\left(v\right)+\sigma_{A}{}^{2}\right)}{\left(MQ_{E_{k}}\left(v\right)+\sigma_{E_{k}}{}^{2}\right)\left(2MQ_{A}\left(v\right)+\sigma_{A}{}^{2}+\sigma_{h_{\mathrm{ab}}}{}^{2}\right)}\\ +\log_{2}\left(\frac{{\left(\sigma_{A}{}^{2}\right)}^{2M-2}}{{\left(\sigma_{A}{}^{2}+\sigma_{h_{\mathrm{ab}}}{}^{2}\right)}^{M-1}{\left(\sigma_{1}{}^{2}/P\right)}^{M}}\right) \end{array}\\ =\log_{2}\frac{f_{k}\left(v\right)}{h_{k}\left(v\right)}+\log_{2}\left(\frac{{\left(\sigma_{A}{}^{2}\right)}^{2M-2}}{{\left(\sigma_{A}{}^{2}+\sigma_{h_{\mathrm{ab}}}{}^{2}\right)}^{M-1}{\left(\sigma_{1}{}^{2}/P\right)}^{M}}\right) \end{array}\;$$

*where*$f_{k}\left(v\right)=M^{3}Q_{A}{\left(v\right)}^{2}Q_{E_{k}}\left(v\right)-M^{3}Q_{A}\left(v\right)Q_{E_{k}}{\left(v\right)}^{2}+2M^{2}\sigma_{A}{}^{2}Q_{A}\left(v\right)Q_{E_{k}}\left(v\right)+$$M^{2}\sigma_{E_{k}}{}^{2}Q_{A}{\left(v\right)}^{2}-M^{2}\sigma_{A}{}^{2}R_{{\mathrm{AE}}_{k}}{\left(v\right)}^{2}+2M\sigma_{A}{}^{2}\sigma_{E_{k}}{}^{2}Q_{A}\left(v\right)+M\sigma_{A}{}^{4}Q_{E_{k}}\left(v\right)+\sigma_{A}{}^{4}\sigma_{E_{k}}{}^{2}$ *and* $h_{k}(v)=2M^{2}Q_{A}\left(v\right)Q_{E_{k}}\left(v\right)+M\left(\sigma_{A}{}^{2}+\sigma_{h_{\mathrm{ab}}}{}^{2}\right)Q_{E_{k}}\left(v\right)+2M\sigma_{E_{k}}{}^{2}Q_{A}\left(v\right)+\left(\sigma_{A}{}^{2}+\sigma_{h_{\mathrm{ab}}}{}^{2}\right)\sigma_{E_{k}}{}^{2}$.

**Proof** **of** **Theorem** **1.** Because the rank of $R_{\mathrm{BS}}$ is 1, it has only one non-zero eigenvalue, which can be denoted as $\lambda_{M}\left(R_{\mathrm{BS}}\right)$, $\lambda_{M}\left(R_{\mathrm{BS}}\right)=\mathrm{tr}\left(R_{\mathrm{BS}}\right)=M$. Therefore, we can have $\sum_{i=1}^{M}\lambda_{i}\left(Q_{A}\left(v\right)R_{\mathrm{BS}}\right)=\mathrm{tr}\left(Q_{A}\left(v\right)R_{\mathrm{BS}}\right)=MQ_{A}\left(v\right)$, and thus we can deduce that the matrix $Q_{A}\left(v\right)R_{\mathrm{BS}}+\sigma_{A}{}^{2}I$ has M−1 identical eigenvalue $\sigma_{A}{}^{2}$, the M eigenvalue is $MQ_{A}\left(v\right)+\sigma_{A}{}^{2}$. Then we utilize the properties that the determinant of a given matrix can be expressed as the product of all eigenvalues of the matrix. Therefore, $\det\left(Q_{A}\left(v\right)R_{\mathrm{BS}}+\sigma_{A}{}^{2}I\right)=\prod_{i=1}^{M}\lambda_{i}\left(Q_{A}\left(v\right)R_{\mathrm{BS}}+\sigma_{A}{}^{2}I\right)={\left(\sigma_{A}{}^{2}\right)}^{M-1}\left(MQ_{A}\left(v\right)+\sigma_{1}{}^{2}\right)$, where $\lambda_{i}\left(\cdot \right)$ means the i-th eigenvalue of the matrix. The determinant of the other three matrix terms can be calculated similarly, which completes the verification. □

We can prove that $C_{k,2}=C_{k,1}$ due to the reciprocal assumption of uplink and downlink.

## 4. Proposed Optimization Scheme and Solution

### 4.1. Optimization Analysis

From the above analysis, we can see the reflection coefficient vector v of IRS leads to the correlation between the legitimate channel and the eavesdropping channel. Therefore, we can design v to maximize the secret key capacity. We can see that key capacity is the minimum secret key capacity $C_{k,1}$ against all the eavesdroppers. A low-complexity optimization scheme is designed to decide the N-element reflecting coefficients of Rose to maximize the key capacity. We can formulate the optimization problem as follows:

(24) $$\begin{array}{ccc} \underset{v}{\max}\underset{k\in K}{\min}C_{k,1} & s.t. & {\left|v_{n}\right|}^{2}\leq 1,n=1,\cdots ,N, \end{array}$$

where K={1,⋯,K}. The function $\log_{2}\left(\cdot \right)$ is monotonically increasing in (23). Accordingly, the optimization formulation (23) can be further described as

(25) $$\underset{v}{\max}\underset{k\in K}{\min}\frac{f_{k}\left(v\right)}{h_{k}\left(v\right)}s.t.{\left|v_{n}\right|}^{2}\leq 1,n=1,\cdots ,N.$$

Due to the max–min operations, the objective function in (25) is non-convex. As described below, we use an auxiliary variable Q to transform the optimization problem as

(26) $$\begin{array}{l} \underset{v,Q}{\max}Q\\ s.t.{\left|v_{n}\right|}^{2}\leq 1,n=1,\cdots ,N\\ Q\in \mathbb{R}\\ Q\cdot h_{k}\left(v\right)-f_{k}\left(v\right)\leq 0,k=1,\cdots ,K. \end{array}$$

It is difficult to find the optimal solution of the problem in (26), because $h_{k}\left(v\right)$ and $f_{k}\left(v\right)$ are both non-convex with respect to the optimization variable v. We denote $V=vv^{H}$, conforming that V≽0 and rank(V)=1. We define $A=\psi^{H}\psi$, $B=\beta^{H}\beta$ and C=ψβ, which are all rank-1 matrices. Since there is non-convexity of the rank-1 constraint, we proceed to apply the SDR technique to relax this constraint. By replacing $v^{H}Xvv^{H}Yvv^{H}Zv=\mathrm{Tr}\left(XVYVZV\right)$, $v^{H}Xvv^{H}Yv=\mathrm{Tr}\left(XVYV\right)$, and $v^{H}Zv=\mathrm{Tr}\left(ZV\right)$, where X,Y,Z∈{A,B,C}, which are any positive semi-definite matrices, and Tr(·) is the trace of a matrix, which is a convex function. We can transform $h_{k}\left(v\right)$ and $f_{k}\left(v\right)$ into function expressions $f_{k}\left(V\right)$ and $h_{k}\left(V\right)$. We can now see that $f_{k}\left(V\right)$ and $h_{k}\left(V\right)$ are both convex with regard to variable V. The optimization problem can be re-formulated as

(27) $$\begin{array}{l} \underset{V,Q}{\max}Q\\ s.t.\mathrm{Tr}\left(E_{n}V\right)\leq 1,V≽0,n=1,\cdots ,N\\ Q\in \mathbb{R}\\ Q\cdot h_{k}\left(V\right)-f_{k}\left(V\right)\leq 0,k=1,\cdots ,K, \end{array}$$

where $E_{n}=e_{n}e_{n}{}^{H}$, $e_{n}$ is a base vector, whose n-th element is 1 and others are 0. So, we have ${\left|v_{n}\right|}^{2}={\left|v^{H}e_{n}\right|}^{2}=v^{H}e_{n}e_{n}{}^{H}v=v^{H}E_{n}v=\mathrm{Tr}\left(E_{n}V\right)\leq 1$. The constraint $Q\cdot h_{k}\left(V\right)-f_{k}\left(V\right)\leq 0$ is the form of the difference of the convex (DC) function for a given Q. In order to further overcome the non-convexity, we propose an efficient sub-optimal solution based on the CCP algorithm, where the Taylor expansion at $V^{\left(q\right)}$ is utilized as

(28) fk∧(V)=fk(V(q))+Tr(Re{∇fk(V(q))H(V−V(q))}).

Therefore, the original constraint condition $Q\cdot h_{k}\left(V\right)-f_{k}\left(V\right)\leq 0$ can be decomposed into multiple convex subproblems by linearizing the concave term $-f_{k}\left(V\right)$. We successfully transformed the IRS optimization problem in (27) into a convex optimization problem, which can be solved by alternatively optimizing Q and V.

Algorithm 1 presents the details of the proposed SDR and CCP based algorithm. The matrix V we obtain is not necessarily rank-1, so we can recover v from V with the help of the Gaussian randomization method. ε denotes a small convergence threshold, and $Q_{\max}$ is a sufficiently large number. Note that the proposed SDR-CCP based algorithm can solve the problem with a worst-case complexity of $O\left(\max{\left\{N,K\right\}}^{4}N^{1/2}\overset{-}{\;L}\log\left(Q_{\max}/\varepsilon \right)\right)$, where $\overset{-}{\;L}$ is the average number of iterations. The SDR-CCP algorithm is more efficient than the standard branch-and-bound algorithm with the exponential complexity of $O\left({\left(1/\delta_{A}\delta_{P}\right)}^{KN}\right)$ in solving problem (27), where $\delta_{A}$ and $\delta_{p}$ are the discrete quantization intervals of amplitude and phase, respectively.
**Algorithm 1** Our algorithm based on SDR and CCP**Input**: $\sigma_{A}{}^{2},\sigma_{E_{k}}{}^{2},\sigma_{h_{\mathrm{ab}}}{}^{2},\eta_{1},\eta_{2},\eta_{3},\beta ,\Psi ,M,\varepsilon ,Q_{\max}$**Output**: V, Q1: Initialize $Q_{\min}=0$, and Set $Q^{\left(t\right)}=\left(Q_{\max}+Q_{\min}\right)/2$.
2: **repeat** (Bisection Search for Q)
3:  **repeat** (Our algorithm based on SDR and CCP)
4:    For given $Q^{\left(t\right)}$, when q=1, initialize a positive semi-definite matrix $V^{\left(1\right)}$ randomly; when
 q>1, given $V^{\left(q\right)}$, solve the above optimization problem (28) for $V^{q+1}$.
5:    Update q=q+1.
6:  **until** the optimization variable V reaches convergence.
7:    Record $V_{opt}=V^{\left(q\right)}$ of the current iteration if the above problem can be solved, then update
$Q_{\min}=Q^{\left(t\right)}$; if $Q^{\left(t\right)}$ is unreachable, then update $Q_{\max}=Q^{\left(t\right)}$
8: **until**
$Q_{\max}-Q_{\min}<\varepsilon$.
9: Recover v from $V_{opt}$ by Gaussian randomization.


### 4.2. Partially Colluding and Fully Colluding Cases

For colluding eavesdropping, all colluding eavesdroppers transmit signals wirelessly to an additional channel signal processor, which also incurs high costs. In the process of wireless transmission, it is easier to expose eavesdroppers’ identities. We consider partially and fully colluding cases, in which the SNR received by the signal processor is the sum of all eavesdroppers’ SNRs. The optimization objective is $\underset{v}{\max}\sum_{k=1}^{X}C_{k,1}$, where X is the number of colluding eavesdroppers. When X=K, it is fully colluding. We solve this optimization problem based on the SDR and successive convex approximation (SCA) methods [24].

### 4.3. Discussion on Active Pilot Attacks

We studied an IRS-assisted key generation scheme against multiple correlated eavesdroppers under the assumption of passive attacks. Next, we discuss the possibility of the proposed scheme to resist a simple active attack, where the eavesdroppers transmit pilots at the same time that the legitimated user is transmitting [27]. In this case, eavesdroppers transmit the same probing signals as those of the legitimated user in the uplink to prevent Alice and Bob from obtaining similar channel estimates. Under this attack, Alice obtains the uplink channel estimation as

(29) $$Z_{a}{}^{UL}=h_{\mathrm{ab}}{}^{T}+G_{b}{}^{T}v+\sum_{k}\left(h_{{\mathrm{ae}}_{k}}{}^{T}+G_{e_{k}}{}^{T}v\right)+N_{a}{}^{{UL}^{\prime }}$$

while in the downlink, Bob estimates the channel as

(30) $$Z_{b}{}^{DL}=h_{\mathrm{ab}}+v^{T}G_{b}+N_{b}{}^{{DL}^{\prime }}$$

From (29) and (30), the channel estimates of Alice and Bob are different, leading to the failure of consistent keys generated. However, as mentioned above, we deploy the IRS to enhance the legitimate user’s channel and deteriorate the channel quality of eavesdroppers. We elaborately design the reflection coefficient matrix of the IRS, greatly weakening the eavesdropping channels. Thus, the eavesdroppers’ signals can be ignored. Alice and Bob can still obtain similar channel estimates and generate secret keys under the pilot spoofing attack. To summarize, the proposed scheme can counteract this attack by elaborately designing the IRS phase shift.

## 5. Simulation Analysis

**Figure 2:**
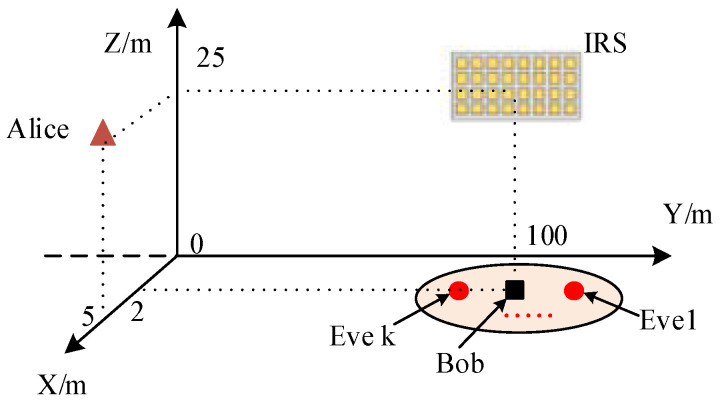
Simulation setup.

As shown in Figure 2, a network deployment where Alice, Bob, and the central point of IRS are located at (5,0,25), (2,100,0), and (0,100,25) is considered. We set up the following simulation parameters according to [17,28]. K Eves are randomly distributed in a circle of radius R centered on Bob. The carrier frequency is set to 1 GHz. IRS configuration is a uniform planar rectangular array (URA) with 5 rows and N/5 columns, and λ/2 spacing. The constant path loss term is ${\mathrm{PL}}_{0}=-20\;\mathrm{dB}$ at reference distance $d_{0}=1\;m$. Since there are extensive obstacles as well as scatters in the channel links from the BS to the user, we set the path loss exponent $c_{\mathrm{ab}}=c_{{\mathrm{ae}}_{k}}=4.5$; meanwhile, we set $c_{\mathrm{ar}}=3.7,\;c_{\mathrm{rb}}=c_{{\mathrm{re}}_{k}}=c_{{\mathrm{be}}_{k}}=2.2$. The other parameters are set as $\sigma_{A}{}^{2}=\sigma_{E_{k}}{}^{2}=-80\;\mathrm{dB}$, $\sigma_{h_{\mathrm{ab}}}{}^{2}=-50\;\mathrm{dB}$, M=4, N=20, K=3, R=λ, and Rician κ-factor κ=1, if not specified otherwise. The maximum correlation coefficient ρ among all Eves is 0.5 unless otherwise specified. Three benchmark schemes are presented: (1) the secret key capacity of key generation from correlated wireless channels without IRS (N-IRS) [29]; (2) an IRS-aided system with on–off switching states (On-off IRS) [19]; (3) an IRS-aided system with random phase shifts (R-IRS) [20].

**Figure 3:**
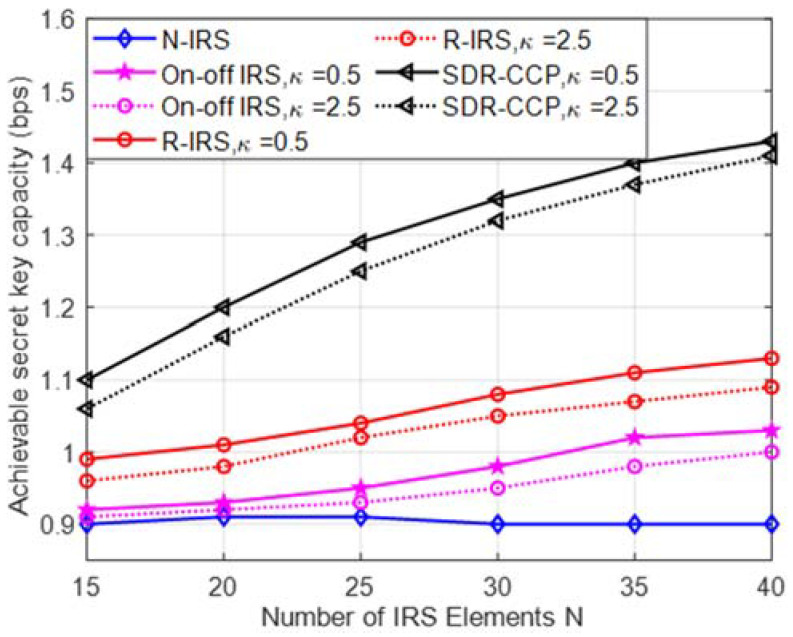
Achievable secret key capacity versus the number of IRS reflecting elements N with P=20dBm .

Figure 3 shows the secret key capacity versus the number of IRS elements N. We see that increasing the size of the IRS results in a significant improvement in the secret key capacity of our SDR-CCP scheme. Furthermore, we observe that our scheme with optimal IRS phase shifting outperforms the three benchmark schemes for the entire range of N, because the SNR of the legitimate user is improved by elaborately designing the phase shift matrix of the IRS. Secret key capacity increases with decreasing Rician κ-factor $\kappa_{\mathrm{rb}}=\kappa_{{\mathrm{re}}_{k}}=\kappa$ because a lower κ corresponding to a stronger NLoS path provides more randomness in the wireless channels.

**Figure 4:**
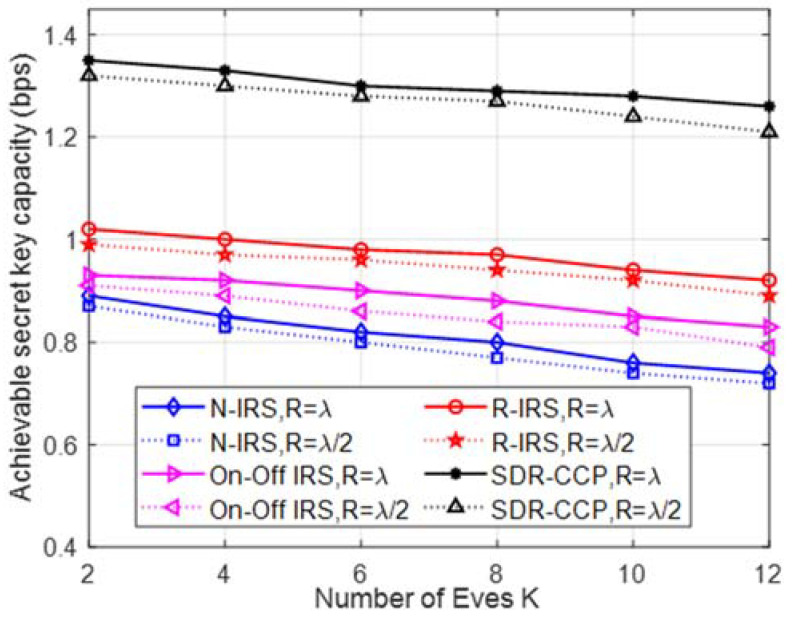
Achievable secret key capacity versus the number of eavesdroppers.

In Figure 4, we note that the achievable secret key capacity reduces with an increasing number of Eves. We also see that our proposed scheme significantly outperforms the three benchmark schemes by at least 0.3 bps. We observe that the increasing number of Eves has only a small negative impact on the performance of our SDR-CCP scheme. As we know, due to a greater average distance between the eavesdroppers with R = λ, the secret key capacity increases.

**Figure 5:**
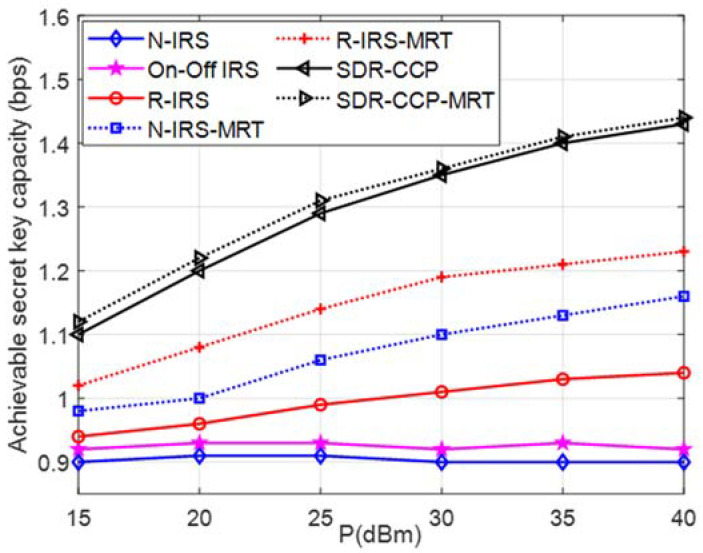
Achievable secret key capacity versus different transmission power P.

In Figure 5, the secret key capacity of our proposed SDR-CCP scheme is significantly improved with the increase of transmission power P, because the channel estimation accuracy at Bob is greatly improved; instead, Eve’s SNR is severely reduced through the design of the IRS phase shift. We consider the MRT precoding strategy used by the transmitting antenna for Bob as a comparison. The MRT scheme without an IRS (N-IRS-MRT) can increase the system’s secret key capacity compared with N-IRS and R-IRS because the base station uses MRT to improve Bob’s SNR. Even if the IRS phase shift is randomly set, MRT (R-IRS-MRT) can improve Bob’s SNR and secret key capacity compared with R-IRS. For the N-IRS and R-IRS, the SNR of Eve is also improved with the increase of P, which increases the risk of information leakage. It can be seen that the increase of secret key capacity based on MRT and SDR-CCP is not significant, because IRS has brought a great increase to Bob’s SNR.

**Figure 6:**
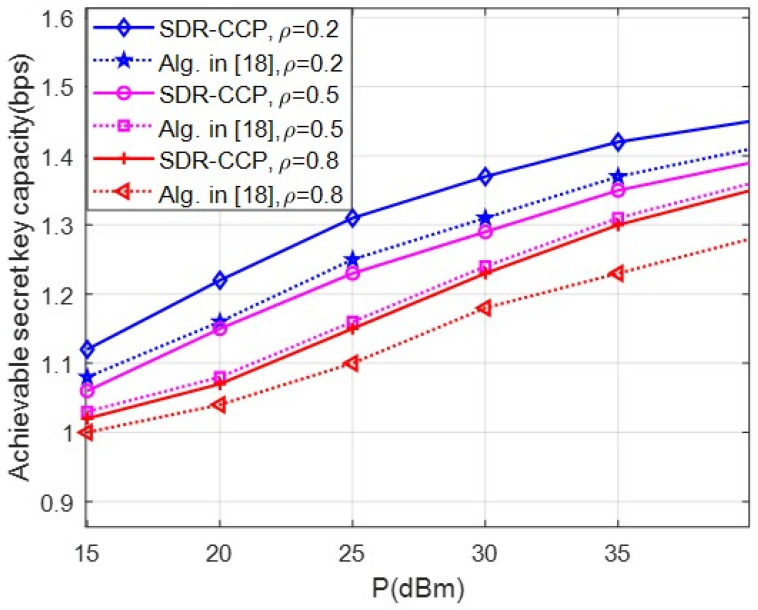
Comparison of the achievable secret key capacity considering correlated or independent eavesdropping for different channel correlation coefficient.

Figure 6 compares the secret key capacity obtained as a function of the channel correlation coefficient ρ. The solutions of the algorithm in [18] are obtained by optimizing the IRS configuration under the assumption of independent channels. Then, the optimal solutions obtained are substituted into the expression of (23). As expected, our SDR-CCP outperforms the algorithm in [18], which assumes independent Eves, in the considered range. In particular, as the channel correlation coefficient increases from 0.2 to 0.8, the key capacity becomes smaller. Figure 6 confirms that the secret key capacity can be enhanced if the channel correlation is considered at the design stage.

**Figure 7:**
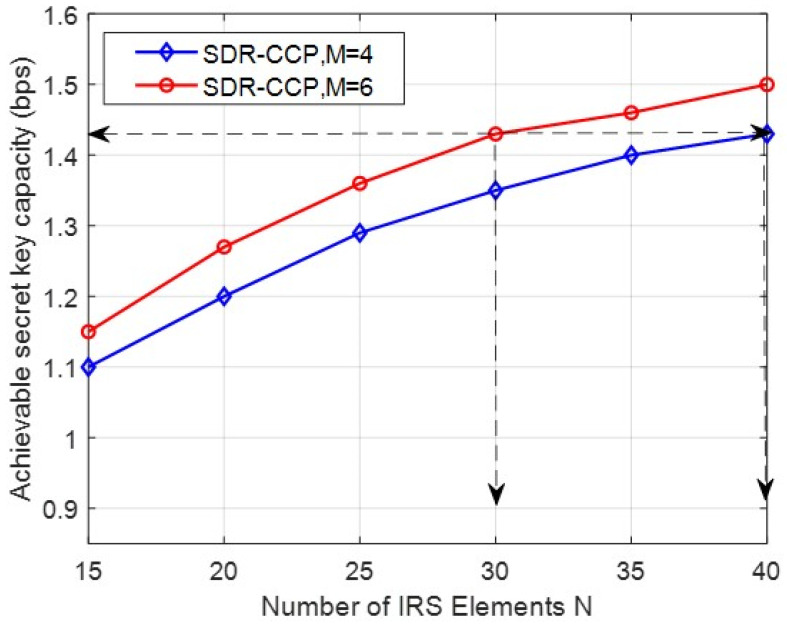
Achievable secret key capacity versus N with different M.

In Figure 7, it is assumed that the transmission power of each antenna is 5dBm. It can be seen that the secret key capacity can be improved by increasing the number of transmitting antennas and IRS elements with our proposed SDR-CCP scheme. For example, the case of M=4, N=40 and M=6, N=30 can achieve the same secret key capacity, which means we can reduce the number of transmitting antennas by increasing IRS elements and reduce the hardware costs of transmitting antennas.

**Figure 8:**
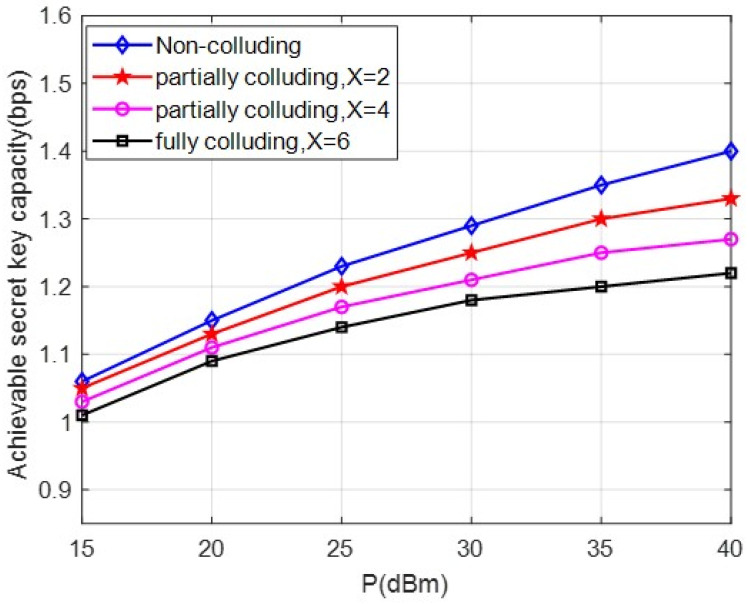
Comparison of the secret key capacity for different numbers of colluding eavesdroppers with different P.

Figure 8 demonstrates the comparison over the non-colluding, partially colluding, and fully colluding cases. We assume K=6 eavesdroppers exist. We consider the influence of the number of colluding eavesdroppers on the secret key capacity in the colluding eavesdropping scenario. It is observed that the risk of leakage becomes larger for a greater number of colluding eavesdroppers, where X = 2,4,6 (fully colluding), which causes a greater security threat than non-colluding eavesdroppers. In particular, the secret key capacity gap becomes more distinct over the non-colluding, partially colluding, and fully colluding cases for higher transmitting power P.

## 6. Conclusions

This paper proposes an IRS-assisted key generation scheme in a MISO system against multiple correlated eavesdroppers. The secret key capacity of the system is maximized by elaborately optimizing the reflection coefficient matrix of IRS. We formulate and solve an optimization problem to obtain the optimal IRS configuration based on SDR and CCP. We find that the same secret key capacity is obtained by reducing the number of transmitting antennas at the cost of increasing the number of IRS elements, which reduced the hardware costs of antennas. The numerical results demonstrated that the proposed scheme provides the highest secret key capacity as compared with other existing benchmark schemes.

## Data Availability

Data are contained within the article.
